# Microwave-assisted synthesis, characterization, and *in vitro* biological evaluation of a novel nanocomposite using molybdenum and [2,2′-bipyridine]-4,4′-dicarboxylic acid

**DOI:** 10.1039/d4ra03758h

**Published:** 2024-08-05

**Authors:** Mohamed J. Saadh, Nadhir N.A. Jafar, Farag M. A. Altalbawy, Pawan Sharma, Abhishek Kumar, Hassan Thoulfikar A. Alamir, Hameed Ghazy, Maha Noori Shakir, Saad khudhur Mohammed, Khursheed Muzammil, Baneen chasib gabal

**Affiliations:** a Faculty of Pharmacy, Middle East University Amman 11831 Jordan; b Al-Zahraa Center for Medical and Pharmaceutical Research Sciences (ZCMRS), University of Al-Zahraa for Women Kerbala 65001 Iraq nather.najim@alzahraa.edu.iq; c Department of Chemistry, University College of Duba, University of Tabuk Tabuk Saudi Arabia; d Department of Chemistry, School of Sciences, Jain (Deemed-to-be) University Bengaluru Karnataka 560069 India; e Department of Sciences, Vivekananda Global University Jaipur Rajasthan 303012 India; f School of Pharmacy-Adarsh Vijendra Institute of Pharmaceutical Sciences, Shobhit University Gangoh Uttar Pradesh 247341 India; g Department of Pharmacy, Arka Jain University Jamshedpur Jharkhand 831001 India; h Department of Pharmaceutics, Faculty of Pharmacy, University of Al-Ameed Iraq; i Department of Pharmacy, Al-Manara College for Medical Sciences Maysan Iraq; j Department of Medical Laboratories Technology, AL-Nisour University College Baghdad Iraq; k Collage of Pharmacy, National University of Science and Technology Dhi Qar 64001 Iraq; l Department of Public Health, College of Applied Medical Sciences, Khamis Mushait Campus, King Khalid University Abha KSA; m Medical Laboratory Technique College, The Islamic University Najaf Iraq; n Medical Laboratory Technique College, The Islamic University of Al Diwaniyah Al Diwaniyah Iraq; o Medical Laboratory Technique College, The Islamic University of Babylon Babylon Iraq

## Abstract

Currently, nanocomposites are synthesized and used in various fields. One of the applications of these nanostructures is in the medical field. Therefore, the synthesis of new composites with biological properties is important. In this study, under microwave conditions, a new nanocomposite containing molybdenum and [2,2′-bipyridine]-4,4′-dicarboxylic acid (Mo/BPDA) was synthesized. The synthesized Mo/BPDA composite was subjected to biological evaluations such as antibacterial and antifungal properties by clinical and laboratory standards institute guidelines, and anticancer properties by MTT method. Characterization and structure characteristics of the Mo/BPDA nanocomposite were evaluated using XRD (X-ray diffraction pattern), FT-IR (Fourier-transform infrared), EDAX (energy-dispersive X-ray), EA (elemental analysis), TGA/DTG (thermogravimetric analysis/differential thermogravimetry), SEM (scanning electron microscopy) and BET (Brunauer–Emmett–Teller) analysis. The results indicated relatively high thermal stability (300 °C), high specific surface area (35 cm^3^ g^−1^) and uniform morphology of the synthesized Mo/BPDA nanocomposite. In antibacterial and antifungal activity, minimum inhibitory concentration (between 2 and 256 μg mL^−1^), minimum bactericidal concentration (between 4 and 128 μg mL^−1^), and minimum fungicidal concentration (between 64 and 256 μg mL^−1^) were tested and reported. The results showed that the antibacterial and antifungal activity of Mo/BPDA nanocomposite is higher than that of antibiotic drugs such as ampicillin, cefazolin, ketoconazole, and nystatin. In the investigation of the anticancer activity that was tested against bone cancer cells and breast cancer cells for 24 and 48 hours, cell proliferation and viability (37.3648–82.0674 tan control) and IC_50_ (33–43 μg mL^−1^) were observed. As a final result, it can be stated that the synthesized Mo/BPDA nanocomposite after the additional biological evaluations, such as *in vivo* study, can be used as an efficient option in treating bone cancer cells and breast cancer cells and a strong antibiotic on a wide range of infectious diseases.

## Abstract 2

Novel Mo/BPDA nanocomposite with crystalline structure, high thermal stability and specific surface area was synthesized. The Mo/BPDA nanocomposite was effective against a wide range of microbial species, bone cancer cells and breast cancer cells.

## Graphical abstract

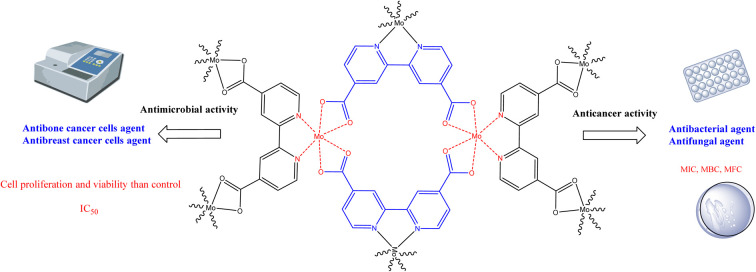

## Introduction

In today's world, nanotechnology and its daily use and applications are undeniable for humanity. Nano compounds have found a special place in our lives due to their unique and interesting properties. Using of nanoparticles in growth of useful plants for humans,^1^ nanopackings in food,^2^ nanomedicines for cancer therapy^3^ and nanofibers in clothes^4^ are some examples. Therefore, researchers have focused on nanotechnology and are devoted to numerous researches. New nano compounds with various properties are synthesized and reported on a routine basis. Nanotechnology has made considerable advances in various fields such as agriculture, environment, basic sciences, engineering, and medicine.

Among the advances in agriculture, we can mention nano-fertilizers, noble metal nanoparticles as antimicrobial agents, and protection of plants against pathogens resistant to multiple drugs.^5^ In this regard, we can mention gold nanoparticles, which also have environmental applications.^6,7^

It can be argued that the most important application of nanotechnology in the environment is the synthesis and reporting of new nano-compounds with unique abilities to absorb and remove environmental pollutants.^8^ Nano zerovalent irons (nZVI) used for the purification of waters and soils and the removal of dyes are some of the most important examples in this field.^9,10^ Removal of azo and anthraquinone reactive dyes from industrial wastewater by magnesium oxide nanoparticles (MgO NPs) is another example of the use of nanoparticles in this field.^11^

A review of the literature shows that MgO NPs have a high ability in the synthesis of heterocyclic and organic compounds.^12–15^ Therefore, it can be mentioned as an example of the application of nanoparticles in organic chemistry, which is classified as basic science. In organic chemistry, synthesis, reporting, and presentation of recyclable and green catalysts have a special place.^16^ The synthesis and reporting of magnetic nanocatalysts is expanding in this field. The magnet core in magnetic catalysts is Fe_2_O_3_.^17,18^

Nano Fe_2_O_3_ is also used in other fields such as the drilling fluid and petroleum industry, which are examples of nanotechnology in engineering sciences.^19,20^ Another application of Fe_2_O_3_ nanoparticles is medical diagnosis such as biosensors.^21,22^

Research on nanotechnology in the field of medicine is very extensive and many studies have been performed in many different fields of this branch of science. In various fields of medical science, such as tissue engineering, biosensing, bioimaging,^23^ and applications such as drug delivery,^24^ treating cancer,^25^ and synthesis of nano compounds with antimicrobial,^26^ antioxidant,^27^ and anticancer properties,^28^ it is a cornerstone for the progress of nanoscience.

Therefore, it can be stated with certainty that research in nanotechnology, including the synthesis of nano-compounds that have valuable properties, can greatly help humanity.

In the field of medicine, nanocomposites, which are one of the most important categories of nano-compounds, have gained a good position. The wound dressings, bone engineering, drug delivery, biosensor applications, antibacterial, antifungal, and anticancer agents are among the reported applications of nanocomposites in medical science.^29–32^ There have been reports of bioactive nanocomposites where metal nanoparticles such as titanium, zinc, silver, copper, molybdenum, and others have been present in their structure.^33–37^

The review of reports shows that molybdenum and nano-compounds containing molybdenum can perform many biological activities. Among these biological activities that can be mentioned are anti-cancer, anti-microbial, antioxidant, anti-tumor and anti-allergic.^38–42^ Therefore, molybdenum can be a suitable option for synthesizing novel bioactive nanocomposites or bioactive nanocomplex using organic ligands. One of the interesting organic compounds that has the ability as ligands to synthesize complexes and nanocomposites is [2,2′-bipyridine]-4,4′-dicarboxylic.

The [2,2′-bipyridine]-4,4′-dicarboxylic acid is an organic compound containing two pyridine rings and two carboxylic acid groups, which are reported in the synthesis of nanocomposites and complexes with dye-sensitized solar cell, redox flow battery, anticancer activity, antipsychotic drugs, DNA/HSA binding affinity and cytotoxic activity, antibacterial and antifungal activity, functional capabilities in the medical industry, *etc.*^43–48^ For example, novel nanocomposite containing [2,2′-bipyridine]-4,4′-dicarboxylic acid named Fe_3_O_4_@SiO_2_@Ru(BiPy)_2_(BPC) were synthesized and reported as DNA recognition.^49^ The complex containing cobalt and [2,2′-bipyridine]-4,4′-dicarboxylic acid was reported with anticancer activity against MCF-7 cancer cell.^50^ The complex containing ruthenium and [2,2′-bipyridine]-4,4′-dicarboxylic with antioxidant activity, DNA/protein interaction and cytotoxicity against HCT-15, HeLa, SKOV3, MCF7 and SKMel2 cell lines is another example of the biological activity of [2,2′-bipyridine]-4,4′-dicarboxylic acid.^51^

As mentioned, molybdenum and [2,2′-bipyridine]-4,4′-dicarboxylic acid have a high ability in biological activities such as antibacterial, antifungal and anticancer. Therefore, by using them, it is possible to synthesize a new nanocomposite that has the biological properties of both. In this research, we synthesized a new nanocomposite using molybdenum(vi) chloride and [2,2′-bipyridine]-4,4′-dicarboxylic (BPDA). As we predicted, the newly synthesized nanocomposite had remarkable biological activities, including antibacterial, anticancer, and antifungal. The observed biological properties were almost equal to some known drugs in the market, and of course, in some cases, they showed better properties than some drugs.

## Results and discussion

### Mo/BPDA structure and characterization

Molybdenum(vi) chloride and BPDA were subjected to the conditions mentioned in synthesis of Mo/BPDA section, including microwave radiation with a power of 350 W for 250 minutes. This radiation can create a temperature close to 120 °C (ref. 52) and lead to the synthesis of a new Mo/BPDA nanocomposite.

**Fig. 1:**
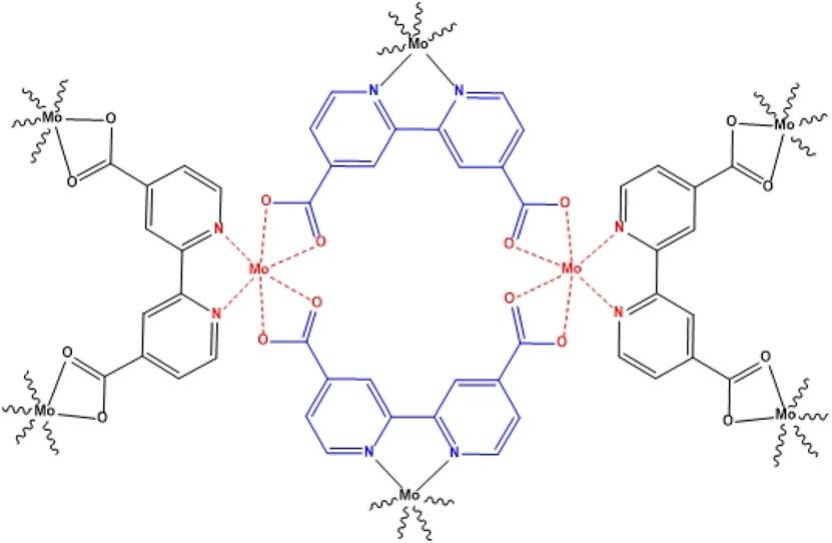
Structure of Mo/BPDA nanocomposite.

Fig. 1 shows the structure proposed for the Mo/BPDA nanocomposite, which was confirmed by XRD, FT-IR, EDAX, and CHNO elemental analysis. The TGA, SEM and BET were other analyses that were used to characterize the Mo/BPDA nanocomposite.

**Fig. 2:**
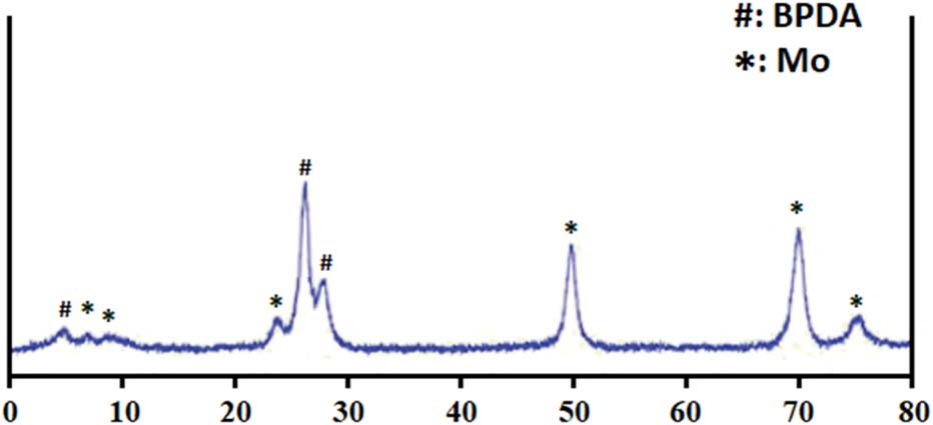
XRD pattern of Mo/BPDA nanocomposite.

As mentioned, the metal used in the synthesis of Mo/BPDA nanocomposite was molybdenum. To obtain the XRD pattern, a 100 mg Mo/BPDA nanocomposite was used. The XRD pattern of the Mo/BPDA (Fig. 2) is similar to the pattern reported previously. According to this pattern, the diffracted peaks in 2 theta angles of 8°, 10°, 24°, 50°, 70° and 75° are related to Mo crystals. Low-intensity peaks near 10° indicate the octahedral structure of the Mo/BPDA nanocomposite.^53–55^ Also, the BPDA showed diffraction peaks in 2 theta angles near 6°, 27°, and 29°.^56^

The crystal structure and the presence of molybdenum in the nanocomposite are proven (Fig. 2). The crystalline structure and nanosize of the Mo/BPDA nanocomposite (75 nm, obtained using the Debye–Scherrer equation)^57^ can be attributed to the synthesis method and the use of microwave radiation.^58,59^ Of course, drying for 3 hours under a vacuum at 25 °C can be effective.

To obtain the SEM image, 500 mg Mo/BPDA nanocomposite was used.

**Fig. 3:**
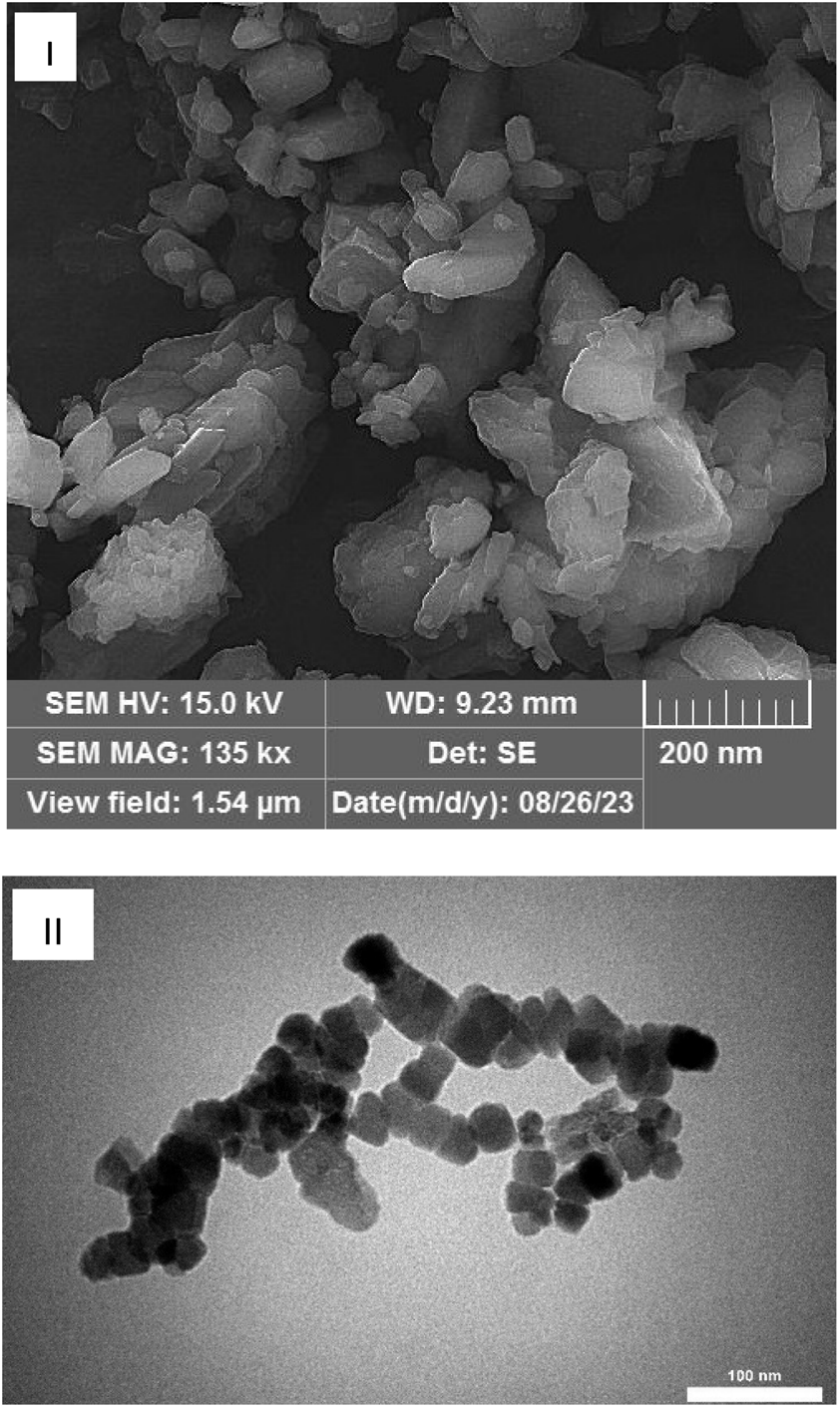
SEM (I) and TEM (II) images of Mo/BPDA nanocomposite.

The SEM image (Fig. 3(I)) of the Mo/BPDA nanocomposite is another proof of the nanostructure of the final product and its identical morphology.

In order to determine the morphology of the Mo/BPDA nanocomposite sample with more focus, the TEM images shown in Fig. 3(II) were obtained.

Based on the obtained results, the formation of the nanocrystals with octahedral morphology was seen. These results are in agreement with the X-ray diffraction pattern that shows octahedral crystals. In addition, the morphology of the Mo/BPDA nanocomposite is uniform, which affects the efficiency of the products.

Based on the obtained results, the formation of the nanocrystals with octahedral morphology was seen. These results are in agreement with the X-ray diffraction pattern that shows octahedral crystals. In addition, the morphology of the Mo/BPDA nanocomposite is uniform, which affects the efficiency of the products.

**Fig. 4:**
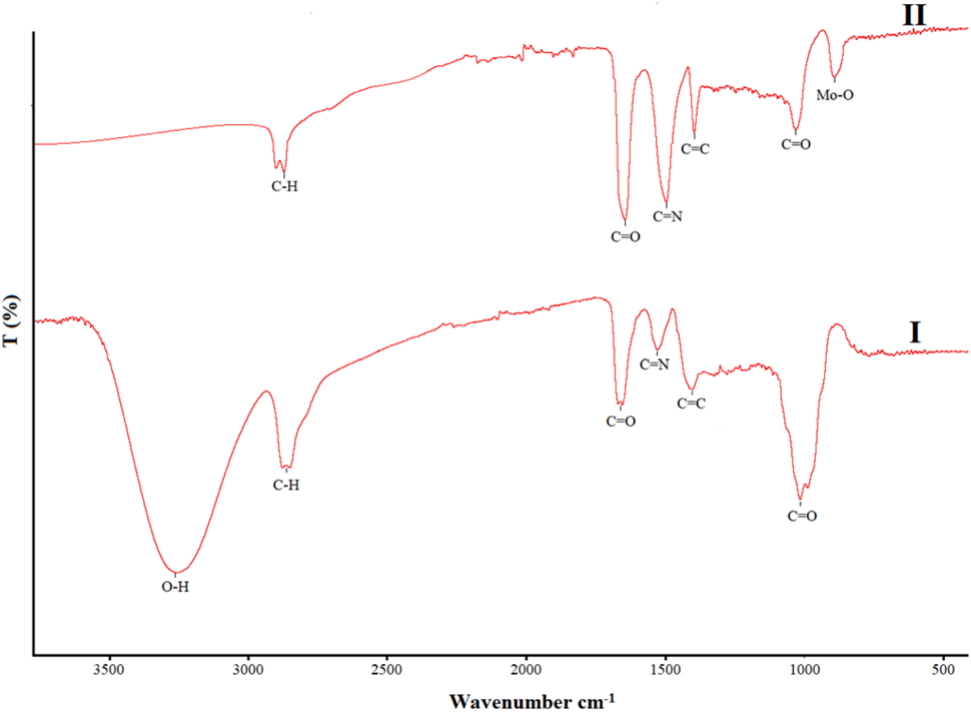
FT-IR spectra of BPDA (I), and Mo/BPDA nanocomposite (II).

The FTIR spectra of BPDA and Mo/BPDA nanocomposite are shown in Fig. 4 for comparison.

In the FTIR spectra of BPDA (Fig. 4(I)) and Mo/BPDA nanocomposite (Fig. 4(II)), absorptions related to C–O (near 1100 cm^−1^), C=C (near 1400 cm^−1^), C=N (near 1500 cm^−1^), C=O (near 1650 cm^−1^) and C–H (near 2900 cm^−1^)^54,60,61^ can be seen in both spectra. Only two essential differences can be seen in these two spectra, which can be used to prove the proposed structure for the final product, as shown in Fig. 1. In the FTIR spectrum of Mo/BPDA nanocomposite, the Mo–O absorption can be seen near 950 cm^−1^ (ref. 54,62) and not observed in FTIR spectra of BPDA. Another difference is related to the O–H bond, which is observed in the FTIR spectra of BPDA (near 3300 cm^−1^) but is not present in the FTIR spectrum of Mo/BPDA.

**Fig. 5:**
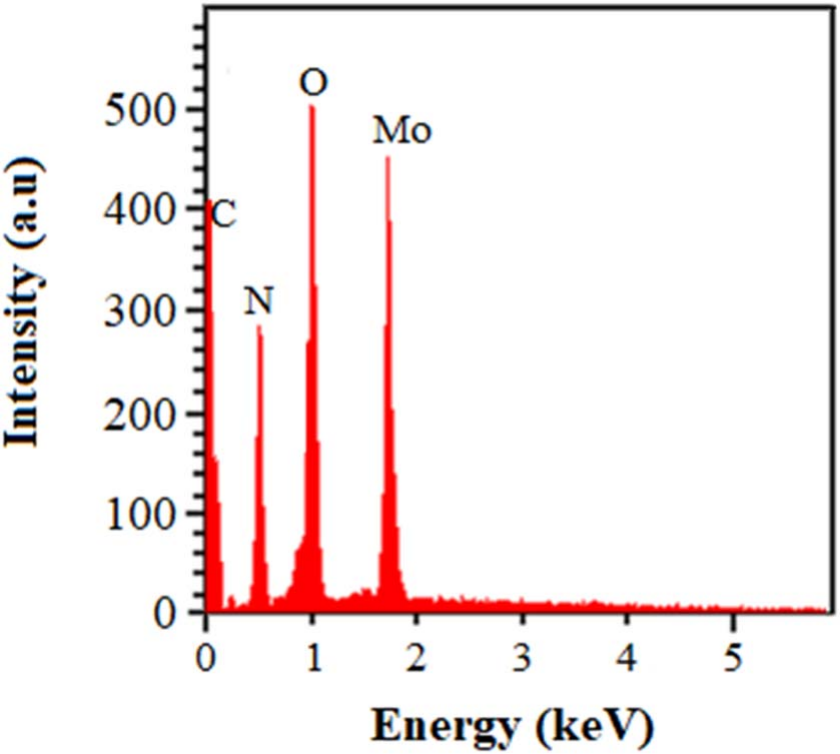
EDAX spectrum of Mo/BPDA nanocomposite.

The EDAX of Mo/BPDA nanocomposite and CHNO elemental analysis of BPDA and Mo/BPDA nanocomposite are given in Fig. 5 and Table 1.

**Table 1:** EA of the Mo/BPDA nanocomposite

Compounds	Criterion	Element (%)					Total percentage (%)
		C	H	N	O	Mo	
BPDA	Calculated	59.02	3.30	11.47	26.21	—	100
	Observed	59.05	3.28	11.44	23.23	—	100
Mo/BPDA nanocomposite	Calculated	52.31	2.68	10.17	23.23	11.61	100
	Observed	52.35	2.66	10.16	23.25	—	88.42

The EDAX Mo/BPDA proves the presence of carbon (C), nitrogen (N), oxygen (O), and molybdenum (MO) in its structure.

The results of Table 1 show that the total percentage of elements in BPDA is equal to 100%, but in the Mo/BPDA nanocomposite, it is about 12% less than 100%. This difference can be attributed to the presence of molybdenum in the Mo/BPDA nanocomposite. In addition, the comparison of the percentage of elements in BPDA and Mo/BPDA nanocomposite shows that the percentage of elements in BPDA is lower compared to that in the Mo/BPDA nanocomposite; for example, the percentage of carbon in BPDA was 59% and in Mo/BPDA nanocomposite it was 52%.

**Fig. 6:**
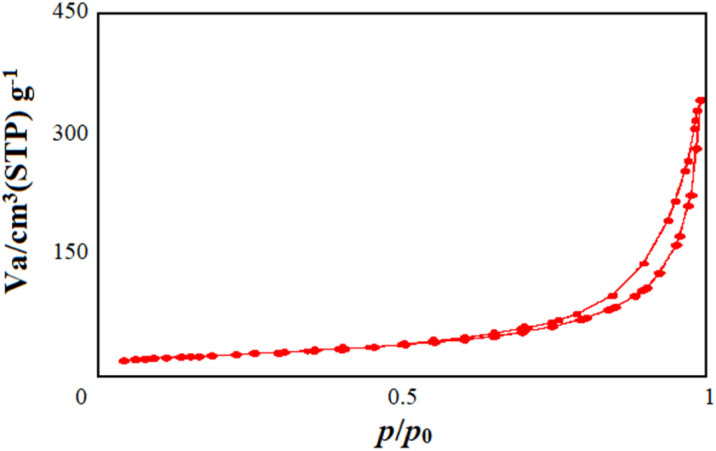
N_2_ adsorption/desorption curves of Mo/BPDA nanocomposite.

The results of N_2_ adsorption/desorption measurements on the synthesized Mo/BPDA nanocomposite (Fig. 6) indicated its high specific surface area.

As shown in Fig. 6, the adsorption/desorption isotherm of the Mo/BPDA nanocomposite was type III. Table 2 shows the Brunauer–Emmett–Teller (BET), Barett–Joyner–Halenda (BJH) pore volume and mean pore diameter (MPD) of the Mo/BPDA nanocomposite. A high specific surface area and high porosity are the factors that lead to increasing the applications and performance of nanoparticles. As discussed in 3–2, the results of these factors and high performance were fully observed in biological assays.

**Table 2:** Brunauer–Emmett–Teller (BET), Barett–Joyner–Halenda (BJH) pore volume and mean pore diameter (MPD) of the Mo/BPDA nanocomposite

BET (m^3^ g^−1^)	BJH (cm^3^ g^−1^)	MPD (nm)
35	0.38	1.27

These parameters also depend on the synthesis method,^63,64^ and the high results obtained in this study also indicate the appropriateness of the synthetic method used.

**Fig. 7:**
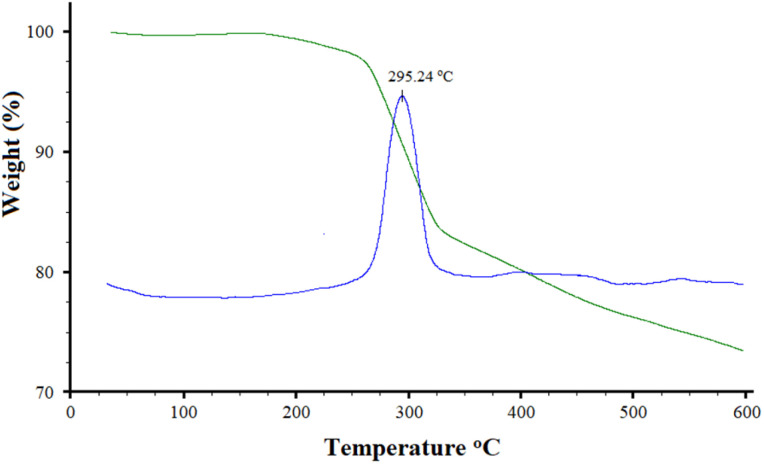
TGA/DTG curves of Mo/BPDA nanocomposite.

Thermal stability, which was performed using TGA/DTG (Fig. 7), was another study performed to characterize the Mo/BPDA nanocomposite.

The TGA/DTG curve of Mo/BPDA nanocomposite showed two specific weight loss. One of which can be attributed to the decomposition of BPDA (near 300 °C) and the other to the destruction of the complex network (350–600 °C). According to the TGA/DTG curve, Mo/BPDA nanocomposite is stable up to 295 °C, a relatively good temperature. Therefore, the synthesized Mo/BPDA nanocomposite has acceptable thermal stability.

### Mo/BPDA biological activity

#### Antibacterial activity of Mo/BPDA nanocomposite

The bacterial strains used in this study were *Edwardsiella tarda* (ATCC 15947), *Klebsiella pneumoniae* (ATCC 13883), *Bacillus cereus* (ATCC 11778), *Shigella dysenteriae* (ATCC 13313), *Streptococcus iniae* (ATCC 29178), *Rhodococcus equi* (ATCC 25729).

To compare the evaluation of antimicrobial activities, tests were performed on molybdenum(vi) chloride, BPDA, and Mo/BPDA, as well as commercial drugs (ampicillin and cefazolin). The results obtained from the tests are given in Table 3.

**Table 3:** Antibacterial activity of the Mo/BPDA nanocomposite^a^

Compounds	Parameters	Species					
		*Edwardsiella tarda*	*Klebsiella pneumoniae*	*Bacillus cereus*	*Shigella dysenteriae*	*Streptococcus iniae*	*Rhodococcus equi*
Molybdenum(vi) chloride	MIC	512	128	512	64	128	64
	MBC	512	256	1024	256	256	128
BPDA	MIC	512	256	—	—	—	512
	MBC	1024	512	—	—	—	1024
Mo/BPDA	MIC	8	2	64	32	32	4
	MBC	16	4	128	32	64	16
Ampicillin	MIC	—	—	—	16	—	8
	MBC	—	—	—	32	—	16
Cefazolin	MIC	4	2	—	—	64	—
	MBC	8	4	—	—	128	—

a MIC and MBC value: μg mL^−1^.

**Fig. 8:**
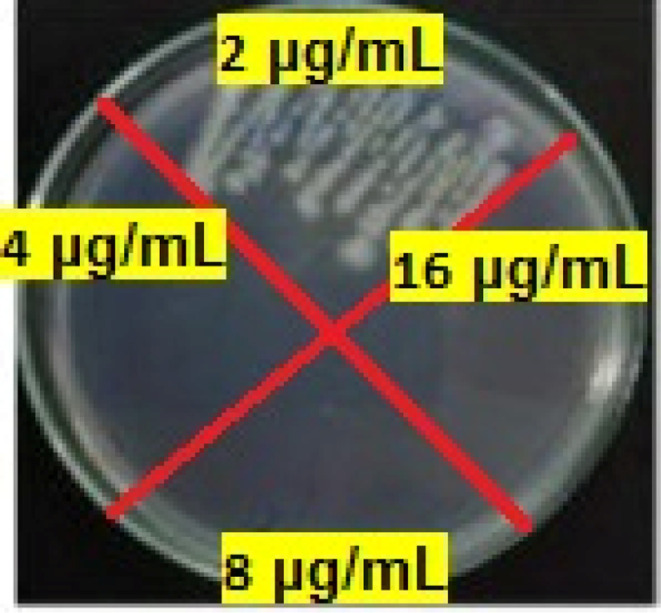
The MBC of Mo/BPDA nanocomposite ageist *Klebsiella pneumoniae*.

The MIC results obtained from the antimicrobial tests of the final product against *Edwardsiella tarda*, *Klebsiella pneumoniae*, *Bacillus cereus*, *Shigella dysenteriae*, *Streptococcus iniae*, *Rhodococcus equi* at 8, 2, 64, 32, 32 and 4 μg mL^−1^, respectively. The MBC results were 16, 4, 128, 32, 64 and 16 μg mL^−1^, respectively. For example, MBC of several concentrations of Mo/BPDA against *Klebsiella pneumoniae* are shown in Fig. 8.

Comparing the results between the raw materials (molybdenum(vi) chloride and BPDA) with the final product (Mo/BPDA), it was observed that the best effectiveness is related to the Mo/BPDA. The obtained result can be attributed to some physical parameters of the final product, such as the nano-size structure and its high specific surface area. Therefore, with the increase of the specific surface area, the contact of the final product with the studied species increases, and, as a result, better effectiveness is achieved.^65,66^ It is crucial to consider the presence of bioactive compounds such as molybdenum in the final product's nanoparticles as one of the main factors.^38–42^

The noteworthy point in the results is that ampicillin is not effective against *Edwardsiella tarda*, *Klebsiella pneumoniae*, *Bacillus cereus*, and *Streptococcus iniae*, and cefazolin is not effective against *Bacillus cereus*, *Shigella dysenteriae*, and *Rhodococcus equi*, but Mo/BPDA is remarkably effective. Therefore, the Mo/BPDA can be a suitable antimicrobial candidate and, as observed, it showed better effectiveness in competition with ampicillin and cefazolin.

#### Antifungal activity of Mo/BPDA nanocomposite

The fungal strains used in this study were *Cryptococcus neoformans* (ATCC 32045), *Candida albicans* (ATCC 10231), *Aspergillus fumigatus Fresenius* (ATCC 1022), *Fusarium oxysporum* (ATCC 7601).

In antifungal evaluations to compare the assessment of antimicrobial activities, tests were performed on molybdenum(vi) chloride, BPDA, and Mo/BPDA as well as commercial drugs (Ketoconazole and Nystatin). The results obtained from the tests are given in Table 4.

**Table 4:** Antifungal activity of the Mo/BPDA nanocomposite^a^

Compounds	Parameters	Species			
		*Cryptococcus neoformans*	*Candida albicans*	*Aspergillus fumigatus*	*Fusarium oxysporum*
Molybdenum(vi) chloride	MIC	256	512	256	512
	MFC	512	1024	512	1024
BPDA	MIC	—	—	512	1024
	MFC	—	—	1024	1024
Mo/BPDA	MIC	256	128	64	64
	MFC	256	256	64	128
Ketoconazole	MIC	—	256	32	256
	MFC	—	512	64	512
Nystatin	MIC	128	—	64	64
	MFC	256	—	128	128

a MIC and MFC value: μg mL^−1^.

**Fig. 9:**
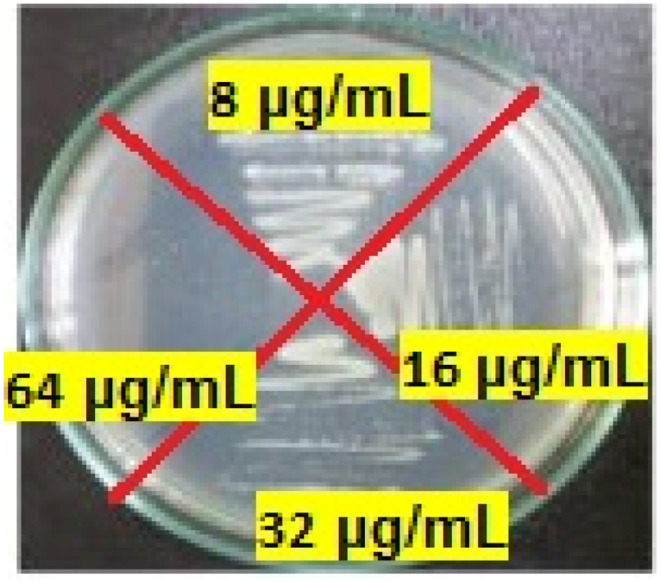
The MFC of Mo/BPDA nanocomposite ageist *Aspergillus fumigatus*.

The MIC results obtained from the antimicrobial tests of the final product against *Cryptococcus neoformans*, *Candida albicans*, *Aspergillus fumigatus Fresenius*, and *Fusarium oxysporum* were 256, 128, 64, and 64 μg mL^−1^, respectively. The MFC results were 256, 256, 64, and 128 μg mL^−1^, respectively. For example, the MFC of several concentrations of Mo/BPDA against *Aspergillus fumigatus* are shown in Fig. 9.

Here is also a comparison of the results between the raw materials (molybdenum(vi) chloride and BPDA) with the final product (Mo/BPDA), it was observed that the best effectiveness is related to Mo/BPDA.

The proposed result of the physical parameters of Mo/BPDA in the high antibacterial activity compared to the raw materials can also be stated in the antifungal activity.^65,66^

The comparison of Mo/BPDA with drugs also proved that ketoconazole is not effective against *Cryptococcus neoformans*, and nystatin is not effective against candida albicans, but Mo/BPDA showed good effectiveness. Therefore, regarding antifungal activity, it can be stated that Mo/BPDA can be introduced as a suitable candidate with acceptable antifungal activity.

#### Anticancer activity of Mo/BPDA nanocomposite

**Fig. 10:**
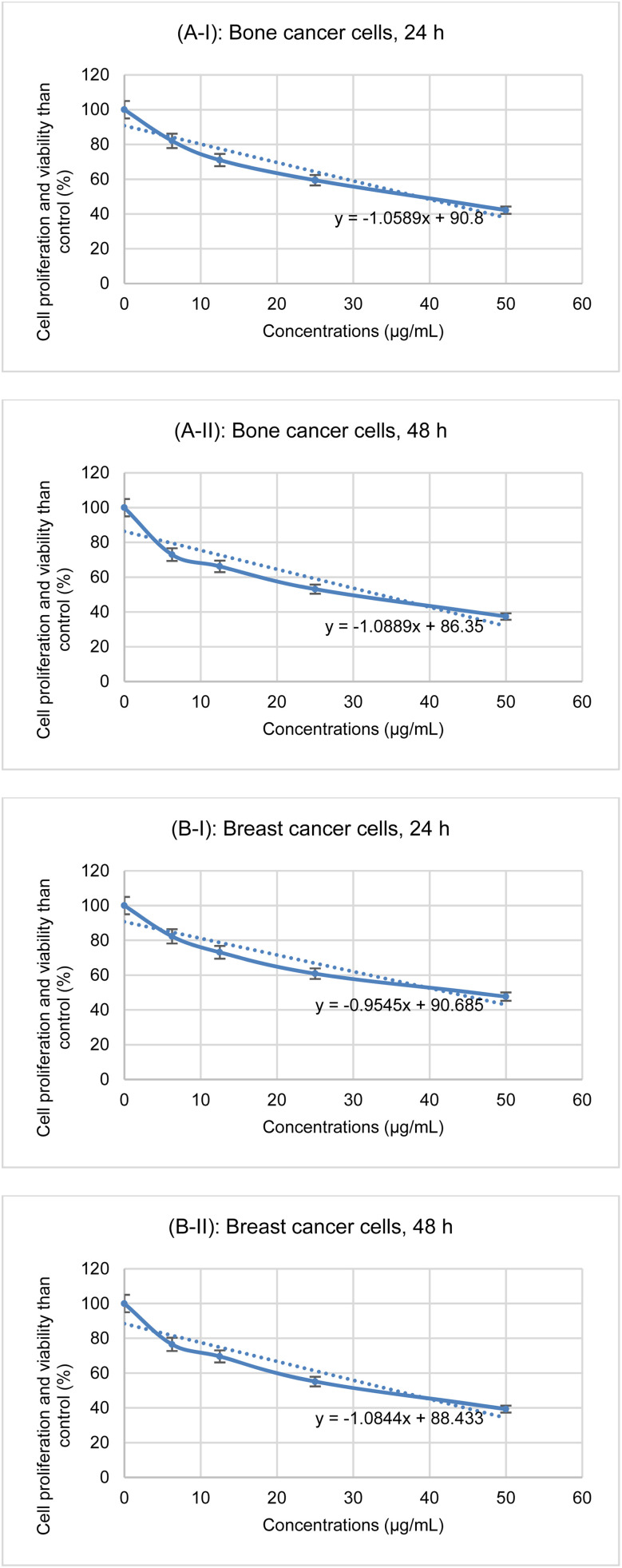
Anticancer activity of Mo/BPDA nanocomposite ((A) bone cancer cells, (B) breast cancer cells).

The anticancer activity of the final product (with concentrations of 6.25 μg mL^−1^, 12.5 μg mL^−1^, 25 μg mL^−1^, and 50 μg mL^−1^) against bone cancer cells (MG-63 – CRL-1427) and breast cancer cells (MCF7 – HTB-22) at 24 h and 48 h were tested (Fig. 10).

In the evaluations, the IC_50_ value was calculated and Fig. 8 shows the obtained line equation that was used to calculate IC_50_.

The results of the anticancer activity including cell proliferation and viability then the control in different concentrations and temperatures and IC_50_ are given in Table 5.

**Table 5:** Anticancer results of the Mo/BPDA nanocomposite

Cancer cells	Time (h)	Concentrations (μg mL^−1^)/cell proliferation and viability than control (%)				IC_50_ (μg mL^−1^)
		6.25	12.5	25	50	
Bone cancer cells	24	82.0674	71.0224	59.4381	42.1973	39
	48	72.9824	66.2047	53.1109	37.3648	33
Breast cancer cells	24	82.3158	73.1249	60.8438	47.6572	43
	48	76.5207	69.5772	55.1249	39.2790	35

The results showed that 50 μg mL^−1^ of Mo/BPDA was the most effective concentration against cancer cells, and 48 hours was the optimal time. In these conditions, the best cell proliferation and viability than the control for bone cancer cells was 37.3648 μg mL^−1^ and for breast cancer cells 39.2790 μg mL^−1^.

The IC_50_ for bone cancer cells at 24 h and 48 h were calculated as 39 μg mL^−1^ and 33 μg mL^−1^, respectively, and for breast cancer cells at 24 h and 48 h were calculated as 43 μg mL^−1^ and 35 μg mL^−1^, respectively.

Statistical studies of IC_50_ at 24 h and 48 h were carried out and the calculated *P*-value are given in Table 6.

**Table 6:** Statistical studies in anticancer activity of the Mo/BPDA nanocomposite

Cancer cell	*P* Value	
	24 h	48 h
Bone cancer cells	0.000	0.002
Breast cancer cells	0.001	0.002

The results proved that the concentration of the final product is the critical parameter at 24 and 48 hours.

Therefore, based on the results obtained from cell proliferation and viability than control, IC_50_, and statistical studies, it can be stated that with the increase in the concentration and time, the final product is more in contact with the cells and leads to their destruction in a significant amount. In anticancer activity as well as antibacterial and antifungal activity studies, the physical properties of the final product that lead to an increase in the contact surface, *i.e.* being nano-sized and having a high specific are surface area, being deduced.^65,66^ It is crucial to consider the presence of bioactive compounds such as molybdenum in the final product's nanoparticles as one of the main factors.^38–42^

## Experimental

### Materials and characterization devices

#### Chemical materials and biological materials

The American Type Culture Collection and Sigma-Aldrich were used to prepare chemicals and bacterial strains. American Type Culture Collection was also the source of the studied cancer cells.

#### Equipment and characterization devices

The equipment and characterization devices used in the analysis of this study are given in Table 7.

**Table 7:** Analysis and characterization using different devices

Analysis	XRD	FT-IR/UV-VIS	EDAX/SEM	EA	TGA	BET
Devices	Shimadzu-XRD-7000	Perkinr-FT-IR UV-VIS pectrum RX1	TESCAN MIRA3	Thermo EA1112	SDT-Q600	BELSORP mini II

#### Synthesis of molybdenum/[2,2′-bipyridine]-4,4′-dicarboxylic acid nanocomposite (Mo/BPDA)

In deionized water (25 mL), dispersed one mmol molybdenum(vi) chloride and one mmol [2,2′-bipyridine]-4,4′-dicarboxylic acid (BPDA) (by ultrasonic treatment). The obtained homogeneous mixture was placed in a microwave reactor with a power of 330 W for 20 minutes.^67^ The synthesized molybdenum/[2,2′-bipyridine]-4,4′-dicarboxylic acid nanocomposite (Mo/BPDA) was isolated by nanofiltration and for purification, first washed three times with deionized water and then three times with ethanol and dried in an oven at 25 °C under vacuum for 3 hours.

### Biological evaluation

#### Antibacterial investigation of Mo/BPDA

The Clinical and Laboratory Standards Institute's standards were followed for measuring minimum inhibitory concentration (MIC), and minimum bactericidal concentration (MBC) when investigating the antibacterial activities of Mo/BPDA nanocomposite. The concentrations of the synthesized Mo/BPDA nanocomposites were between 1 μg mL^−1^ and 2048 μg mL^−1^. The tests were carried out on 1 × 10^5^ CFU mL^−1^ concentration of the studied strains. For the MIC study, 90 μL of the Mueller–Hinton broth, 10 μL of bacterial strain, and 100 μL microliters of Mo/BPDA nanocomposites (a separate concentration in each plate well) were poured into 96 well plates and shaken for 48 h at 37 °C in an incubator. The lowest concentration at which the mixture was clear and had no turbidity was reported as MIC. For the MBC study, the contents of the 96-well plate used in MIC were cultured separately on Mueller–Hinton agar and incubated for 72 h at 37 °C. The lowest concentration in which bacteria did not grow was reported as MBC.^68,69^

#### Antifungal investigation of Mo/BPDA

The Clinical and Laboratory Standards Institute's standards were followed for measuring minimum inhibitory concentration (MIC), and minimum fungicidal concentration (MFC) when investigating the antifungal activities of Mo/BPDA nanocomposite. Concentrations of the synthesized Mo/BPDA nanocomposites were between 1 μg mL^−1^ and 2048 μg mL^−1^. The tests were carried out on 1 × 10^5^ CFU mL^−1^ concentration of the studied strains. For the MIC study, 90 μL of the Sabouraud-Dextrose broth, 10 μL of fungi strain, and 100 μL microliters of Mo/BPDA nanocomposites (a separate concentration in each plate well) were poured into 96 well plates and shaken for 48 h at 27 °C in an incubator. The lowest concentration at which the mixture was clear and had no turbidity was reported as MIC. For the MFC study, the contents of the 96-well plate used in MIC were cultured separately on Sabouraud-Dextrose agar and incubated for 72 h at 27 °C. The lowest concentration in which fungi did not grow was reported as MFC.^68,69^

#### Anticancer investigation of Mo/BPDA

The measurement of the cell proliferation and viability of control, and IC_50_ was carried out and reported based on the MTT methods in the investigation of the anticancer activities of the Mo/BPDA nanocomposite. At concentrations of 6.25 μg mL^−1^ to 50 μg mL^−1^, synthesized Mo/BPDA nanocomposites were tested and studied. The tests were carried out at 24 and 48 hours on bone cancer cells (MG-63 – CRL-1427) and breast cancer cells (MCF7 – HTB-22).^70^ In the evaluations, the passage number of cells was 3 and the concentration of cells was 1.2 × 10^4^ cells per well.

## Conclusions

The new nanocomposite was synthesized using molybdenum(vi) chloride and BPDA under microwave conditions. XRD, FT-IR, EDAX, CHNO elemental analysis, TGA, SEM, and BET were used to identify and confirm the structure of the newly synthesized Mo/BPDA nanocomposite. The antibacterial, antifungal, and anticancer activities of the synthesized Mo/BPDA nanocomposite were tested. MIC, MBC in antimicrobial activity and IC_50_ in anticancer activity were tested and reported. In the anticancer activity, investigations were carried out on bone cancer cells and breast cancer cells, and IC_50_ was 33 μg mL^−1^ for the bone cancer cells and 35 μg mL^−1^ for the breast cancer cells. Regarding antibacterial and antifungal activity, the effectiveness of the synthesized Mo/BPDA nanocomposite on some of the studied strains was higher than that of ampicillin, cefazolin, ketoconazole, and nystatin. The high efficacy of the synthesized Mo/BPDA nanocomposite can be attributed to some of its unique physical and chemical characteristics, such as the presence of bioactive compounds in its structure, as well as its nanosize and high specific surface area. The newly synthesized Mo/BPDA nanocomposite is capable of being introduced as a highly bioactive candidate with antibacterial, antifungal, and anticancer properties as a final result.

## Data availability

The data supporting the findings of this study are available within the article.

## Author contributions

MJS: writing – original draft, methodology; NNAJ: writing – original draft, writing – review and editing, project administration; FMAA: writing – original draft, methodology; PS: writing – original draft, data curation; AK: writing – review and editing, conceptualization; HTAA: writing – review and editing, formal analysis; HG: writing – review and editing, resources; MNS: writing – original draft, validation; SKM: writing – review and editing, investigation; KM: writing – review and editing, visualization; BCG: writing – review and editing, supervision.

## Conflicts of interest

There are no conflicts to declare.
